# Renin and 1-year mortality in critically ill patients with ARDS: trajectories, discrimination, and survival analysis

**DOI:** 10.3389/fmed.2026.1806797

**Published:** 2026-06-23

**Authors:** Gabriele Melegari, Alessandro Belletti, Federica Arturi, Antonio Giansante, Arianna Gaspari, Fabio Gazzotti, Elisabetta Bertellini, Giovanni Landoni, Alberto Barbieri

**Affiliations:** 1 Surgical Medical and Dental Department of Morphological Sciences related to Transplant, Oncology and Regenerative Medicine, University Hospital of Modena, Modena, Italy; 2 Anaesthesia and Intensive Care, Azienda Ospedaliero-Universitaria di Modena, Modena, Italy; 3 Department of Anesthesia and Intensive Care, IRCCS San Raffaele Scientific Institute, Milan, Italy; 4 School of Anaesthesia and Intensive Care, Universita degli Studi di Modena e Reggio Emilia, Modena, Italy; 5 Esercito Italiano, Rome, Italy; 6 School of Medicine, Vita-Salute San Raffaele University, Milan, Italy

**Keywords:** biomarker, COVID-19, intensive care, long-term outcome, mortality, RAAS, renin, risk stratification

## Abstract

**Background:**

We investigated whether early and serial plasma renin concentration (PRC) is associated with 1-year mortality in critically ill patients with COVID-19-related acute respiratory distress syndrome (ARDS).

**Methods:**

We conducted a single-center retrospective cohort study in a tertiary intensive care unit, including exclusively patients with COVID-19-related ARDS. PRC was measured at 72 h (T0), 120 h (T1), and 168 h (T2) after intensive care unit (ICU) admission. Chronic renin–angiotensin–aldosterone system (RAAS) inhibitors (ACEi/ARB) were discontinued on admission. The primary endpoint was 1-year mortality.

**Results:**

The analysis set included 104 mechanically ventilated patients with COVID-19-related ARDS and available 1-year outcome; 48/104 (46%) died within one year. Higher renin was associated with 1-year mortality at T0 (OR 1.94; *p* = 0.002), T1 (OR 1.69; *p* = 0.014), and T2 (OR 2.52; *p* < 0.001). Kaplan–Meier curves by renin tertiles showed significant separation at T0 and T2 (log-rank *p* < 0.001). In multivariable logistic regression, independent predictors were renin at T0 (adjusted OR 5.15; 95% CI 1.34–19.78; *p* = 0.017), PaO₂/FiO₂ ratio (P/F) at T0 (OR 0.02; *p* = 0.010), and procalcitonin (PCT) at T2 (OR 81.89; *p* = 0.020). Model discrimination was high (AUC 0.941).

**Conclusion:**

In patients with severe COVID-19-related ARDS, early renin (72 h) is independently associated with 1-year mortality, and serial trajectories provide additional prognostic information. Prospective multicenter validation is warranted.

## Introduction

Lactate is currently the most widely used biomarker for assessing disease severity and mortality risk in critically ill patients. Elevated lactate levels are generally viewed as indicators of impaired tissue perfusion and altered cellular metabolism, and they have been consistently linked to poor outcomes (1–4). However, lactate is a nonspecific biomarker; its concentration can be affected by several factors, including adrenergic stimulation, liver dysfunction, and therapeutic interventions, even in the absence of overt circulatory failure.

Similarly, other commonly used biomarkers in the intensive care setting, such as procalcitonin and C-reactive protein, mainly reflect inflammatory or infectious processes (5). Although they are useful for diagnosis and short-term risk stratification, their ability to capture the complexity of prolonged critical illness and evolving organ dysfunction remains limited, particularly when long-term outcomes are considered.

Increasing attention has therefore been directed toward biomarkers that reflect sustained pathophysiological stress and chronic organ dysfunction, as these mechanisms may play a key role in determining long-term prognosis. In this context, the renin–angiotensin–aldosterone system (RAAS) represents a central regulator of vascular tone, renal perfusion, and systemic hemodynamic homeostasis. Persistent activation of this system has been associated with adverse outcomes in conditions characterized by chronic circulatory and organ dysfunction (6, 7).

Plasma renin concentration may thus provide additional prognostic information beyond traditional biomarkers, especially when evaluated serially over time (8, 9). This concept is supported by recent studies highlighting the relevance of biomarkers related to chronic organ dysfunction in predicting long-term mortality across different clinical settings. Since 2012, several studies suggested that renin may be a promising tissue perfusion and resuscitation biomarker in critically ill and perioperative patients (10–14). However, data on long-term prognostic impact of renin remains limited, and no study specifically focused on patients with acute respiratory distress syndrome (ARDS). We therefore performed a retrospective study to assess the correlation between renin kinetics and 1-year mortality in critically ill patients with ARDS.

### Aim of the study

The aim of the study is to assess whether plasma renin measured at 72 h (T0), 120 h (T1), and 168 h (T2) after ICU admission is associated with 1-year mortality in ARDS patients, and to demonstrate whether renin trajectories enhance risk stratification beyond clinical severity scores and the PaO₂/FiO₂ ratio.

## Methods

### Ethics statement

This retrospective observational study was approved by the local Ethics Committee (Comitato Etico Area Vasta Modena, protocol 784/21) and conducted in accordance with the Declaration of Helsinki. The requirement for written informed consent was waived due to the retrospective design of the study, in accordance with Italian data-protection regulations. The study included patients admitted to the ICU of Baggiovara Civil Hospital, Azienda Ospedaliero–Universitaria di Modena (Italy), from 29 February 2020 to June 2021.

### Inclusion criteria

We included all critically ill coronavirus disease 2019 (COVID-19) patients who fulfilled the following criteria: confirmed SARS-CoV-2 infection by polymerase chain reaction (PCR) from a nasopharyngeal swab; diagnosis of COVID-19-related ARDS; at least one chest CT scan performed after ICU admission; requirement for invasive mechanical ventilation; and age ≥18 years. COVID-19-related ARDS was defined according to the Berlin definition. Exclusion criteria included: no radiologic evidence of COVID-19 pneumonia at ICU admission and pregnancy. Serial renin sampling was performed per protocol feasibility; complete-case subsets were defined for specific analyses.

### Patient management

Patient management in the intensive care unit followed contemporaneous national and international recommendations as well as local institutional protocols, which evolved over time during the COVID-19 pandemic. Sedation was managed according to standard ICU practice, with propofol used as the first-line agent and midazolam introduced when prolonged or deeper sedation was required. Respiratory care adhered to ARDS principles, including lung-protective ventilation, individualized PEEP, prone positioning when indicated, and short courses of neuromuscular blockade when required. Hemodynamic management was based on a conservative fluid strategy and the use of vasopressors to maintain adequate perfusion. Norepinephrine was the first-line agent to achieve a target mean arterial pressure ≥65 mmHg, with additional vasoactive support, including epinephrine, administered when necessary. In cases of suspected or documented cardiac dysfunction, inotropic agents such as dobutamine or levosimendan were considered based on clinical judgment, with serial perfusion assessment guiding therapy. Empirical antibiotic therapy was commonly initiated at ICU admission due to the high risk of bacterial superinfection, and subsequently tailored according to clinical evolution, microbiological data when available, and antimicrobial stewardship principles in line with current guidelines.

Diuretic therapy was considered in patients with reduced urine output (<0.5 mL/kg/h), as part of standard fluid management strategies. Given the unprecedented nature of the pandemic, clinical protocols were highly dynamic and subject to continuous updates over time. Therefore, while general management principles were consistent, some variability in therapeutic approaches across patients cannot be excluded.

Chronic angiotensin converting enzyme inhibitor/angiotensin receptor blocker (ACEi/ARB) therapy was suspended on ICU admission for COVID-19. Chronic renin–angiotensin–aldosterone system inhibitors (ACE inhibitors and angiotensin receptor blockers) were discontinued upon ICU admission in all patients, in accordance with institutional practice during the COVID-19 pandemic. Given the acute critical illness setting and the short half-life of circulating renin, no predefined washout period was required, and renin measurements were interpreted as reflecting disease-driven RAAS activation rather than residual pharmacological effects. Acute kidney injury was managed per standard practice, including CVVH when indicated. Antimicrobials were targeted to suspected/confirmed bacterial infection. Standard bundles addressed thromboprophylaxis, stress-ulcer prophylaxis, analgesia/sedation, delirium prevention, early enteral nutrition, and glycemic control. Extracorporeal membrane oxygenation (ECMO) candidacy was considered for refractory hypoxemia.

### Sample measurement

Plasma renin concentration (PRC) was measured at 72 h (T0), 120 h (T1), and 168 h (T2) after ICU admission; these timepoints reflected laboratory assay availability rather than continuous sampling. Renin was measured as direct renin concentration (DRC) using a chemiluminescent immunoassay that quantifies active renin in plasma. Results are expressed in μU/mL, corresponding to assay-specific units of active renin. Blood samples were processed according to standard laboratory procedures, including prompt centrifugation and controlled storage conditions. Repeated freeze–thaw cycles were avoided. Renin was measured using a direct immunoassay designed to quantify active renin, with minimal cross-reactivity with prorenin according to manufacturer specifications. The selected sampling timepoints (72, 120, and 168 h after ICU admission) reflected laboratory assay availability and standardized morning blood sampling schedules during the study period, rather than event-driven or outcome-dependent measurements; ICU admission was therefore selected as the common temporal reference, as the timing of endotracheal intubation and sepsis onset was highly heterogeneous and not reliably comparable across patients in this retrospective cohort. This pragmatic approach ensured consistency and reproducibility across patients while acknowledging the constraints inherent to a retrospective design. At the same timepoints, we recorded vasopressor therapy, renal-replacement therapy (RRT), ECMO, MAP (synchronized with the morning arterial blood gas), PaO₂/FiO₂ (P/F) ratio, lactate, C-reactive protein (CRP), procalcitonin (PCT), and Sequential Organ Failure Assessment (SOFA) Score (in addition to admission scores). Procalcitonin was included as a routinely available biomarker reflecting late inflammatory or infectious burden; detailed microbiological data on secondary infections were not systematically available. Vital status at 6 months and 1 year has been retrieved from electronic registries; the primary endpoint was 1-year mortality, with 6-month mortality as a secondary endpoint. Time-to-event data were used to describe the timing of mortality. For descriptive purposes, deaths were classified as early (≤30 days), intermediate (31–90 days), or late (>90 days) after ICU admission. Additional analyses were performed to enhance interpretability and are reported in Supplementary material (including correlations and univariable regression models across timepoints and supplementary outcome analyses).

### Statistical analysis

During the study period, analyses were run in STATA 16 and GraphPad Prism. ChatGPT Plus was used only to help draft and format the text, not for data handling or inference. Continuous variables are shown as mean ± standard deviation (SD). Since some biomarkers were skewed (e.g., renin and procalcitonin), we formally assessed their distributions; when markedly non-normal, median (IQR) values were inspected and inferential analyses were performed on log-transformed variables. Mean ± SD was retained as the primary descriptive summary to ensure consistency and comparability across tables, while all regression analyses relied on log-transformed biomarkers to improve robustness and reduce the impact of skewness (15). Categorical variables are reported as *n* (%) and compared with *χ*^2^ (or Fisher’s exact when expected counts <5). Serial measures at T0 (72 h), T1 (120 h), T2 (168 h) were compared cross-sectionally at each timepoint with the same univariable tests, using available cases per variable/timepoint (so denominators may differ). All tests were two-sided with *α* = 0.05; no multiplicity correction was applied at this descriptive stage. For longitudinal trends (Renin, Lactate, CRP, PCT), we modeled log(1 + *x*) values with a quadratic time term and a group-by-time interaction (group = 1-year survival). Time was centered at 120 h; patient clustering was handled with cluster-robust SEs. At each timepoint we reported the adjusted difference between groups on the log scale and the corresponding geometric-mean ratio (non-survivors/survivors), with Wald *p*-values. Curves on the original scale were obtained by back-transforming model means and shown with bootstrap 95% confidence intervals (CIs). With only three timepoints per patient, this simple non-linear model was preferred over spline-based approaches.

Univariable associations with 1-year mortality were assessed using logistic regression, entering one predictor at a time. Continuous variables were z-standardized; skewed biomarkers were analyzed on the log(1 + *x*) scale. Results are odds ratios (OR) per 1 SD for continuous predictors and OR (yes vs. no) for binary predictors, with 95% CIs and two-sided *p*-values. Predictors with *p* ≤ 0.20 at screening entered a multivariable logistic model (maximum likelihood on complete cases). We report adjusted ORs (per 1 SD for continuous), 95% CIs, and Wald p-values; multicollinearity was checked and, when scores were redundant, we favored a parsimonious specification. Model discrimination was assessed by ROC/AUC; the Youden threshold is reported with sensitivity and specificity (shown in the LROC figure). No multiplicity or optimism correction was applied at this exploratory stage.

Survival was analyzed with Kaplan–Meier curves using “Time Surv” (days) and 1 year death as the event (1 = event; 0 = censored). Renin at T0/T1/T2 was split into tertiles (Low/Mid/High); groups were compared using two-sided log-rank tests (reported for High vs. Low). Because T1–T2 are post-baseline measures, these plots are descriptive and may be affected by immortal-time bias; time-dependent or landmark analyses are planned as sensitivity checks. *Post hoc* analysis and power size were performed and explained in Supplementary materials.

## Results

During the study period, 249 adults with COVID-19 were admitted for their first ICU stay. The present analysis focuses on the 104 patients with complete 1-year outcome and serial biomarker data (analysis set 104/249 patients). Within this analysis set, 48/104 (46%) died within one year. Baseline characteristics included: mean age 68.5 ± 9.4 years, 71% male, 70% with hypertension, and 33% chronically exposed to ACEi/ARB. Compared with survivors, non-survivors were older (71.4 vs. 66.0 years; *p* = 0.003), had a higher Charlson index (4.1 vs. 2.8; *p* = 0.001), and presented with greater severity by SOFA (7.2 vs. 5.2; *p* = 0.002) and SAPS II (45.8 vs. 39.6; *p* = 0.005) scores, whereas APACHE II did not differ (*p* = 0.159). Sex, hypertension, and ACEi/ARB exposure were not significantly associated with 1-year mortality (all *p* ≥ 0.146). On serial assessment, plasma renin was higher in non-survivors at T0 and T2 (T0: 100.3 vs. 46.8; *p* = 0.020; T2: 86.0 vs. 31.6; *p* < 0.001), while lactate and procalcitonin were also higher at T2 (lactate 1.67 vs. 1.30 mmol/L; *p* = 0.003; PCT 1.32 vs. 0.40 ng/mL; *p* = 0.007). The P/F ratio was lower in non-survivors at T0 (130.9 vs. 174.0 mmHg; *p* = 0.017), and MAP was lower at T1 (83.2 vs. 91.6 mmHg; p = 0.007). Vasopressor use was more frequent at T1/T2 among non-survivors (*p* = 0.012 and *p* = 0.001). Among non-survivors (*n* = 48), 33 patients (68.8%) died within 30 days from ICU admission, 11 (22.9%) between 31 and 90 days, and 4 (8.3%) after 90 days, indicating that most fatal events occurred early during the disease course; serial SOFA scores and organ-support variables further characterized clinical severity and progression according to survival status and timing of death.

Detailed descriptive statistics are reported in Tables 1–4; all figures refer to the analysis set (*n* = 104). To further describe clinical course and timing of mortality, non-survivors were stratified according to early (≤30 days), intermediate (31–90 days), and late (>90 days) death. Serial SOFA scores and organ-support variables differed significantly between survivors and non-survivors, while subgroup analyses by timing of death were descriptive (Table 4).

**Table 1:** Baseline characteristics according to 1-year survival status.

Variable	Overall (*n* = 104)	Survivors (*n* = 56)	Non-survivors (*n* = 48)	*p*-value
Hypertension, *n* (%)	73 (70.2)	36 (64.3)	37 (77.1)	0.227
ACEI/ARB therapy, *n* (%)	34 (32.7)	17 (30.4)	17 (35.4)	0.735
Charlson index, mean ± SD	3.4 ± 1.9	2.8 ± 1.4	4.1 ± 2.3	0.001
Age, years	68.5 ± 9.4	66.0 ± 9.0	71.4 ± 9.2	0.003
Male sex, *n* (%)	74 (71.2)	36 (64.3)	38 (79.2)	0.146
SAPS II at admission	42.5 ± 11.1	39.6 ± 9.5	45.8 ± 12.0	0.005
APACHE II at admission	13.8 ± 6.1	13.0 ± 5.7	14.7 ± 6.5	0.159
SOFA at admission	6.1 ± 3.2	5.2 ± 2.8	7.2 ± 3.3	0.002

Baseline comorbidities relevant to RAAS modulation, including diabetes mellitus, chronic kidney disease, chronic liver disease, and heart failure, were captured as part of the Charlson comorbidity index and are reported in Table 1.

**Table 2:** Biomarkers.

Variable	Overall (*n* = 104)	Survivors (*n* = 56)	Non-survivors (*n* = 48)	*p*-value
Renin T0 (72 h), μU/mL	71.5 ± 113.7	46.8 ± 87.8	100.3 ± 133.1	0.020
Renin T1 (120 h), μU/mL	59.2 ± 95.2	54.0 ± 107.0	65.2 ± 80.1	0.547
Renin T2 (168 h), μU/mL	56.7 ± 74.8	31.6 ± 44.7	86.0 ± 91.0	<0.001
Lactate T0 (72 h), mmol/L	1.50 ± 1.41	1.37 ± 1.24	1.66 ± 1.58	0.302
Lactate T1 (120 h), mmol/L	1.42 ± 0.50	1.34 ± 0.53	1.51 ± 0.46	0.072
Lactate T2 (168 h), mmol/L	1.47 ± 0.64	1.30 ± 0.56	1.67 ± 0.68	0.003
CRP T0 (mg/dL)	13.1 ± 8.8	13.0 ± 9.9	13.3 ± 7.4	0.830
CRP T1 (mg/dL)	9.5 ± 7.7	8.2 ± 7.5	11.0 ± 7.7	0.064
CRP T2 (mg/dL)	7.4 ± 8.8	6.4 ± 9.3	8.7 ± 8.1	0.174
Procalcitonin T0 (ng/mL)	1.44 ± 3.37	1.26 ± 3.67	1.63 ± 3.05	0.587
Procalcitonin T1 (ng/mL)	1.17 ± 2.53	0.78 ± 1.71	1.63 ± 3.19	0.105
Procalcitonin T2 (ng/mL)	0.83 ± 1.65	0.40 ± 0.76	1.32 ± 2.19	0.007

**Table 3:** Organ function and organ support.

Variable	Overall (*n* = 104)	Survivors (*n* = 56)	Non-survivors (*n* = 48)	*p*-value
P/F ratio T0 (mmHg)	154.1 ± 94.0	174.0 ± 101.9	130.9 ± 78.8	0.017
P/F ratio T1 (mmHg)	174.9 ± 74.7	183.7 ± 77.1	163.6 ± 70.9	0.204
P/F ratio T2 (mmHg)	178.0 ± 74.5	188.9 ± 78.2	164.3 ± 68.0	0.118
MAP T0 (mmHg)	91.4 ± 17.2	93.1 ± 17.9	89.6 ± 16.4	0.302
MAP T1 (mmHg)	87.7 ± 16.3	91.6 ± 17.8	83.2 ± 13.2	0.007
MAP T2 (mmHg)	85.2 ± 15.0	85.9 ± 15.2	84.3 ± 14.8	0.586
Vasopressors T0 (72 h), *n* (%)	30 (28.8)	12 (21.4)	18 (37.5)	0.113
Vasopressors T1 (120 h), *n* (%)	26 (25.0)	8 (14.3)	18 (37.5)	0.012
Vasopressors T2 (168 h), *n* (%)	25 (24.0)	6 (10.7)	19 (39.6)	0.001
CVVH T0 (72 h), *n* (%)	2 (1.9)	1 (1.8)	1 (2.1)	1.000
CVVH T1 (120 h), *n* (%)	4 (3.8)	1 (1.8)	3 (6.2)	0.504
CVVH T2 (168 h), *n* (%)	5 (4.8)	2 (3.6)	3 (6.2)	0.860
Antibiotics at T0 *n* (%)	79 (76.00)	40 (71.4)	39 (81.3)	0.261
Antibiotics at T1 *n* (%)	75 (72.1)	37 (66.1)	38 (79.2)	0.188
Antibiotics at T2 *n* (%)	65 (62.5)	29 (51.8)	36 (75.0)	0.016
Tocilizumab at T0 *n* (%)	32 (30.8)	21 (37.5)	11 (22.9)	0.137
Tocilizumab at T1 *n* (%)	2 (1.9)	2 (3.6)	0 (0.0)	0.498
Tocilizumab at T2 *n* (%)	0 (0.0)	0 (0.0)	0 (0.0)	N/A
Steroids at T0 *n* (%)	82 (78.8)	45 (80.4)	37 (77.1)	0.811
Steroids at T1 *n* (%)	98 (94.2)	53 (94.6)	45 (93.8)	1.000
Steroids at T2 *n* (%)	98 (94.2)	51 (91.1)	47 (97.9)	0.214
ECMO (any time), *n* (%)	0 (0.0)	0 (0.0)	0 (0.0)	—
SOFA T1 (120 h)	6.1 ± 3.3	5.0 ± 3.0	7.4 ± 3.2	<0.001
SOFA T2 (168 h)	6.2 ± 3.3	4.9 ± 2.3	7.8 ± 3.7	<0.001

**Table 4:** Clinical course and timing of mortality according to 1-year survival

Variable	Survivors (*n* = 56)	Early ≤30 d (*n* = 33)	31–90 d (*n* = 11)	>90 d (*n* = 4)	*p*-value
SOFA at 72 h (T0)	5.2 ± 2.8	7.4 ± 3.2	6.9 ± 3.4	6.5 ± 3.1	0.002
SOFA at 120 h (T1)	5.0 ± 3.0	7.8 ± 3.1	7.2 ± 3.3	6.8 ± 2.9	<0.001
SOFA at 168 h (T2)	4.9 ± 2.3	8.1 ± 3.6	7.5 ± 3.8	7.0 ± 3.5	<0.001
Vasopressors any time	19/56 (33.9%)	21/33 (63.6%)	6/11 (54.5%)	1/4 (25.0%)	0.018
RRT (CVVH) any time	2/56 (3.6%)	2/33 (6.1%)	1/11 (9.1%)	0/4 (0.0%)	0.660

*p*-values refer to comparisons between survivors and all non-survivors. Values for non-survivors stratified by timing of death are descriptive only; no formal statistical comparisons were performed among these subgroups due to limited sample size. Tables 1–4: Continuous variables are presented as mean ± standard deviation and were compared with Welch’s *t*-test. Categorical variables are presented as counts (percentages) and were compared with the *χ*^2^ test or Fisher’s exact test when appropriate. Serial measurements (T0 = 72 h, T1 = 120 h, T2 = 168 h) were analysed cross-sectionally at each timepoint using available cases; denominators may vary across rows. ACEi/ARB, angiotensin-converting enzyme inhibitors/angiotensin receptor blockers; APACHE II, Acute Physiology and Chronic Health Evaluation II; CRP, C-reactive protein; CVVH, continuous venovenous hemofiltration; ECMO, extracorporeal membrane oxygenation; MAP, mean arterial pressure; PCT, procalcitonin; P/F, PaO₂/FiO₂ ratio; PRC, plasma renin concentration; SAPS II, Simplified Acute Physiology Score II; SOFA, Sequential Organ Failure Assessment. Units: P/F and MAP are expressed in mmHg; CRP in mg/dL; PCT in ng/mL; lactate in mmol/L; PRC reported in laboratory units as per the local assay.

**Figure 1:**
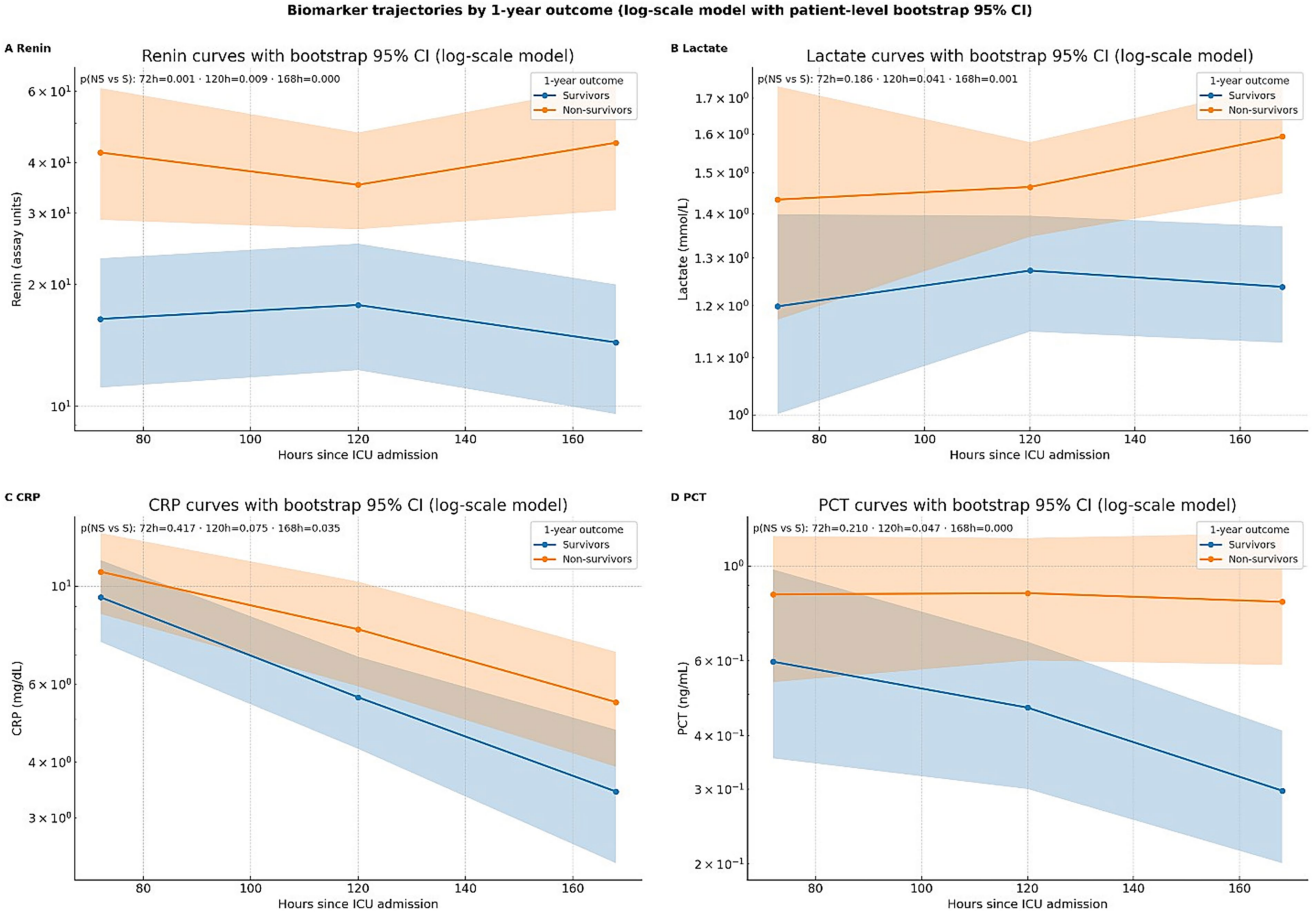
**(A–D)** Biomarker trajectories for Renin **(A)**, Lactate **(B)**, CRP **(C)**, and PCT **(D)** in ICU patients stratified by 1-year survival. Points/lines show model-based means on the original scale obtained from a log-transformed quadratic-time model with patient-level cluster-robust inference; ribbons denote patient-level bootstrap 95% confidence intervals. The *y*-axis is logarithmic. Annotated *p*-values refer to timepoint-specific contrasts (non-survivors vs. survivors) at 72 h, 120 h, and 168 h. See Supplementary Table 1 for model coefficients and confidence intervals.

In longitudinal analyses using a log-quadratic time model with patient-level cluster-robust inference, renin showed significant differences between groups at T0 (72 h) and T2 (168 h) (geometric-mean ratios Non-survivors/Survivors ≈ 1.28, *p* = 0.047; and 1.42, *p* < 0.001, respectively), whereas the contrast at T1 (120 h) was smaller and not significant. These estimates reflect different modeling approaches: logistic regression (per 1-SD increase) and longitudinal models (geometric mean ratios), and are therefore not directly comparable. Lactate and PCT showed higher levels among non-survivors at T2, while CRP differences were not statistically significant across timepoints. Predicted curves with bootstrap 95% CIs are shown in Figure 1; detailed contrasts (log-differences, ratios, and CIs) are reported in Supplementary Table 1.

**Figure 2:**
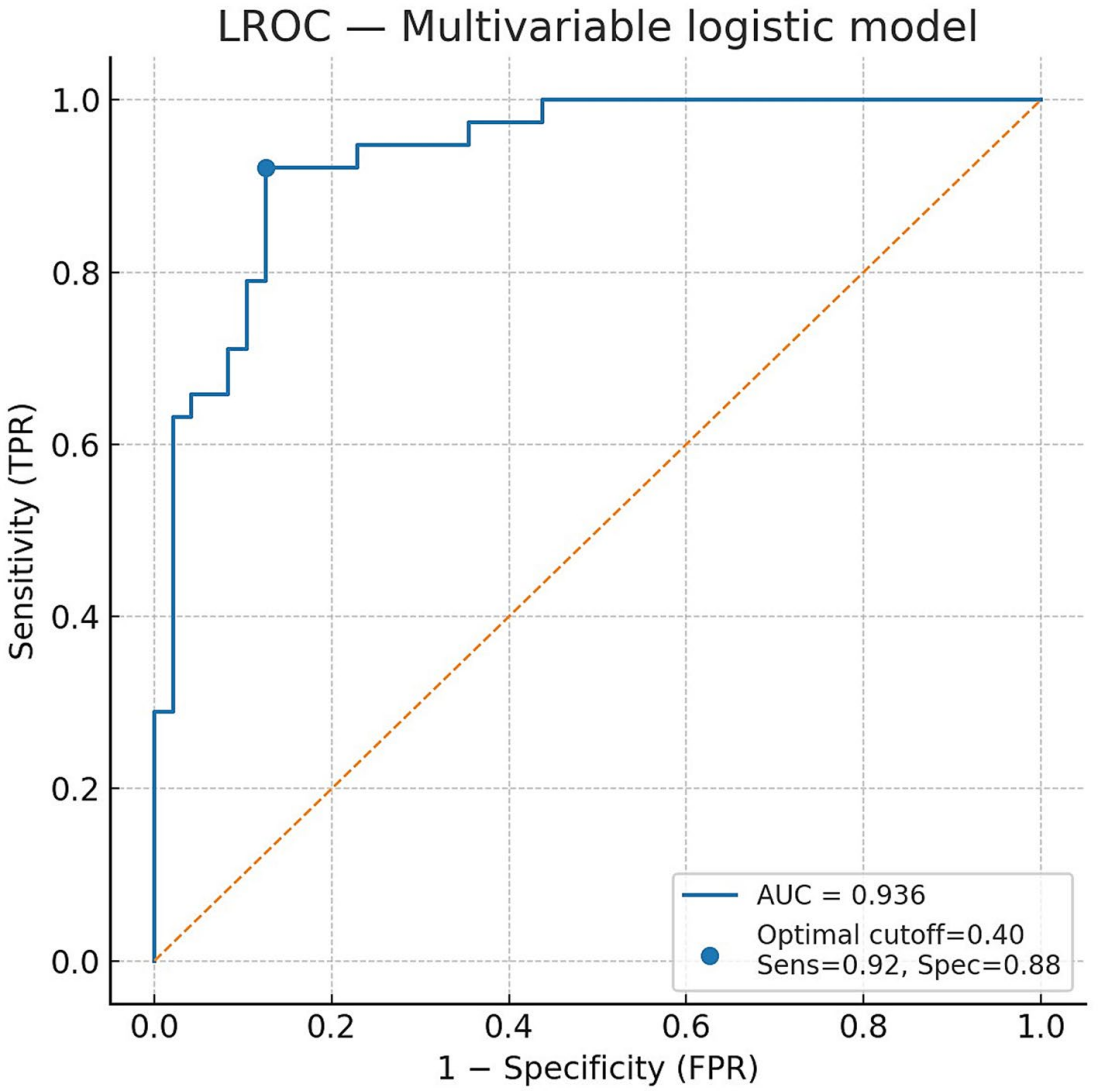
LROC—Multivariable logistic model for 1-year mortality. ROC curve for the conventional multivariable logistic regression fitted on complete cases (*n* = 86, deaths = 38). Candidate predictors were those with univariable *p* ≤ 0.20; continuous predictors were *z*-standardized and skewed biomarkers were analyzed on the log(1 + *x*) scale. The solid curve shows sensitivity vs. 1 − specificity; the dashed diagonal indicates no discrimination. The dot marks the optimal probability threshold by Youden’s index (0.424), yielding sensitivity 0.92 and specificity 0.88. AUC = 0.941. Performance values are apparent (no optimism correction).

At univariable screening (logistic regression, one predictor at a time), older age, higher Charlson index, higher admission SAPS II and SOFA, a lower P/F at T0, higher Renin (T0 and T2), higher Lactate and PCT at T2, lower MAP at T1, and vasopressor use at T2 were associated with 1-year mortality, whereas sex, hypertension, and chronic ACEi/ARB exposure were not (Table 5). Variables with *p* ≤ 0.20 proceeded to multivariable modeling. In the multivariable logistic model (complete cases *n* = 86, deaths = 38), three predictors remained independently associated with mortality—effects expressed as adjusted ORs per 1-SD increase for continuous variables, with biomarkers analyzed on the log(1 + *x*) scale: P/F at T0 (OR 0.02, 95% CI 0.00–0.43; *p* = 0.01), Renin at T0 (OR 5.14, 95% CI 1.34–19.78; *p* = 0.02), and PCT at T2 (OR 81.89, 95% CI 2.02–3320.46; *p* = 0.02). The Charlson index showed a borderline association (OR 2.79, 95% CI 0.98–7.91; *p* = 0.05), and vasopressors at T1 showed a nonsignificant trend (*p* = 0.09; Table 6). Model discrimination was high with an AUC of 0.941 on the ROC curve; the optimal probability threshold by Youden’s index was 0.424, yielding sensitivity 0.92 and specificity 0.88 (LROC) (Figure 2). Given the sample size and number of predictors, these results may be optimistic and will be revisited with parsimonious modeling and internal validation.

**Table 5:** Univariate logistic regression for 1-year mortality (per 1 SD for continuous variables).

Variable	*N*	Odds ratio	95% CI low	95% CI high	*p*-value
ACEi/ARB (chronic)	104	1.258	0.553	2.860	0.584
Age (years)	104	1.914	1.219	3.006	0.005
Charlson index	104	2.226	1.341	3.694	0.002
Hypertension	104	1.869	0.785	4.447	0.158
Sex (male)	104	2.111	0.871	5.118	0.098
APACHE II (admission)	104	1.333	0.894	1.989	0.159
SAPS II (admission)	104	1.887	1.189	2.994	0.007
SOFA (admission)	104	1.936	1.255	2.985	0.003
CRP T0	104	1.175	0.794	1.738	0.419
CRP T1	104	1.434	0.959	2.142	0.079
CRP T2	104	1.523	1.022	2.272	0.039
Lactate T0	104	1.313	0.874	1.972	0.190
Lactate T1	104	1.510	1.000	2.281	0.050
Lactate T2	104	1.982	1.263	3.112	0.003
PCT T0	97	1.301	0.859	1.970	0.214
PCT T1	102	1.528	0.994	2.348	0.053
PCT T2	103	2.361	1.370	4.069	0.002
Renin T0	104	1.944	1.269	2.980	0.002
Renin T1	104	1.687	1.113	2.558	0.014
Renin T2	104	2.516	1.570	4.033	0.000
MAP T0	104	0.815	0.551	1.204	0.303
MAP T1	104	0.522	0.315	0.864	0.011
MAP T2	104	0.897	0.608	1.323	0.583
P/F T0	104	0.567	0.342	0.940	0.028
P/F T1	89	0.755	0.487	1.170	0.208
P/F T2	88	0.701	0.444	1.108	0.129
CVVH T0	104	1.170	0.071	19.226	0.912
CVVH T1	104	3.667	0.369	36.472	0.268
CVVH T2	104	1.800	0.288	11.248	0.530
ECMO T0	104	N/A	N/A	N/A	N/A
ECMO T1	104	N/A	N/A	N/A	N/A
ECMO T2	104	N/A	N/A	N/A	N/A
Vasopressors T0	104	2.200	0.926	5.227	0.074
Vasopressors T1	104	3.600	1.393	9.305	0.008
Vasopressors T2	104	5.460	1.958	15.225	0.001
SOFA T1	104	2.236	1.413	3.537	0.001
SOFA T2	104	2.948	1.725	5.036	0.000

**Table 6:** Multivariable logistic regression (sorted by *p*-value).

Variable	Adjusted OR	95% CI low	95% CI high	*p*-value
P/F T0	0.020	0.001	0.434	0.013
Renin T0	5.145	1.338	19.778	0.017
PCT T2	81.890	2.020	3,320.459	0.020
Charlson Index	2.785	0.980	7.913	0.054
Vasopressors T1	0.042	0.001	1.721	0.094
Sex (male)	12.806	0.492	333.268	0.125
CRP T1	5.495	0.616	49.045	0.127
Renin T2	2.542	0.683	9.461	0.164
CRP T2	0.187	0.015	2.301	0.191
Lactate T1	0.434	0.115	1.628	0.216
SOFA T1	3.410	0.471	24.687	0.225
P/F T2	2.173	0.602	7.848	0.236
SAPS II (admission)	0.388	0.074	2.031	0.262
Age (years)	2.039	0.524	7.930	0.304
APACHE II (admission)	0.468	0.101	2.159	0.330
PCT T1	0.434	0.079	2.375	0.336
MAP T1	0.688	0.222	2.133	0.518
Lactate T0	0.593	0.114	3.078	0.534
Renin T1	0.672	0.132	3.421	0.632
Vasopressors T2	2.313	0.070	76.265	0.638
Vasopressors T0	2.298	0.058	90.634	0.657
Lactate T2	0.831	0.270	2.559	0.747
SOFA T2	1.321	0.141	12.413	0.807
SOFA (admission)	0.889	0.165	4.804	0.892
Hypertension	1.039	0.134	8.065	0.971

Predictors selected by univariate screening (*p* ≤ 0.20). Variables are standardized (continuous per 1 SD; binary 1 vs. 0). Sorted by ascending *p*-value.

**Figure 3:**
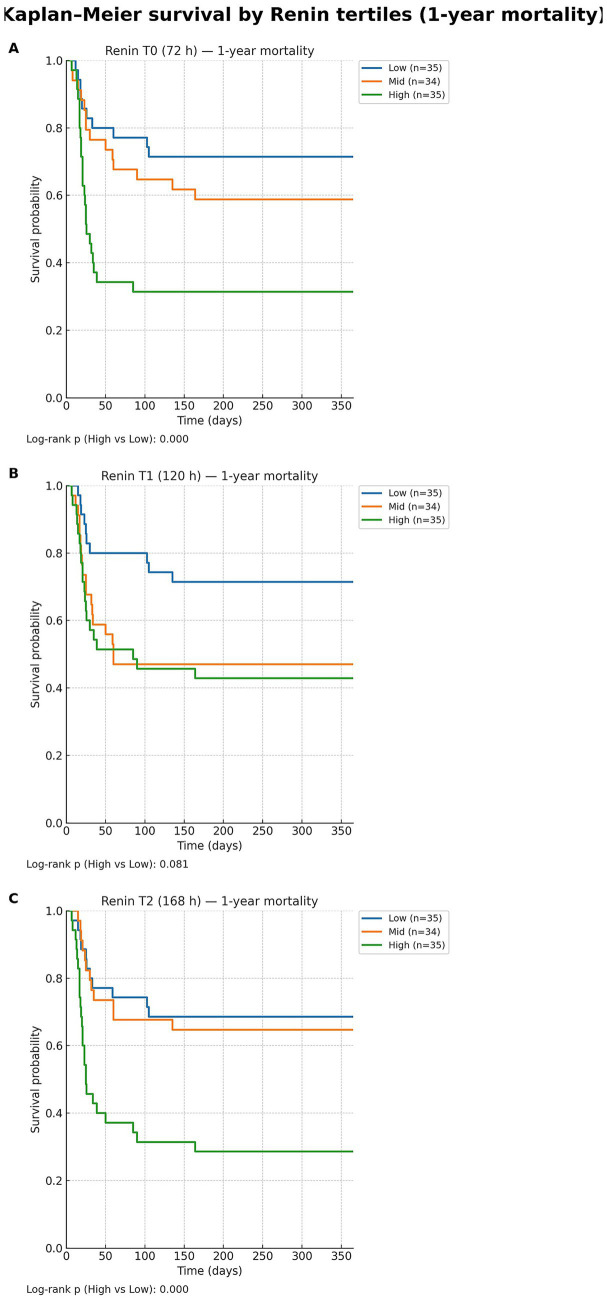
Kaplan–Meier survival by renin tertiles at **(Panel A)** T0 (72 h), **(Panel B)** T1 (120 h), and **(Panel C)** T2 (168 h) for 1-year mortality. Curves extend to 365 days; two-sided log-rank *p*-values are reported for high vs. low tertiles on each panel. Renin tertiles were defined separately at each timepoint using data-driven tertiles. The corresponding ranges were: T0 (72 h) low ≤11.3 μU/mL, intermediate 11.6–39.7 μU/mL, high ≥40.2 μU/mL; T1 (120 h) low ≤11.6 μU/mL, intermediate 11.9–45.2 μU/mL, high ≥46.1 μU/mL; and T2 (168 h) low ≤13.7 μU/mL, intermediate 14.2–55.4 μU/mL, high ≥64.9 μU/mL.

Kaplan–Meier analyses for 1-year mortality (curves extended to 365 days) showed a clear, dose–response pattern across renin tertiles. At T0 (72 h), patients in the High tertile had markedly lower survival than those in the Low tertile (log-rank *p* < 0.001), with the Mid tertile intermediate. At T1 (120 h), the separation persisted as a borderline trend (*p* ≈ 0.081), and at T2 (168 h), the difference was again significant (*p* < 0.001). Because T1–T2 reflect post-baseline measurements, these KM comparisons are primarily descriptive and may be influenced by immortal-time bias; the strong association observed at T0 supports an early prognostic role for renin (Figure 3; Timepoint in Supplemental material). Sensitivity analyses using log-transformed biomarkers yielded consistent results.

## Discussion

In this study, we evaluated the longitudinal behavior of plasma renin concentrations in critically ill patients with COVID-19 admitted to the ICU. Our findings demonstrate that renin levels were markedly elevated at ICU admission (T0), in line with previous evidence suggesting that activation of the renin–angiotensin system reflects systemic hypoperfusion and stress response in critical illness (14, 16–18). Importantly, we observed a heterogeneous temporal trend: while renin levels tended to decrease in most patients during the initial phase (T1), a subsequent increase was observed at later stages (T2) in patients who did not survive. This dynamic pattern suggests that, although renin may initially decline even in severely ill patients, reaching values comparable to those of survivors, the subsequent rebound reflects the persistence of underlying disease severity and a reduced responsiveness to therapeutic interventions. Thus, persistently high or secondarily increasing renin levels appear to identify patients with more advanced organ dysfunction and poor prognosis (19). In our study, the association between renin and 1-year mortality shows a clear risk gradient already at 72 h (T0), with a marked separation of survival curves by tertiles (high vs. low: log-rank *p* < 0.001). At 120 h (T1) the separation is borderline, to then become significant again at 168 h (T2). This pattern supports both the early prognostic value at T0 and the utility of categorical classification when a universal cut-off is not available, given that absolute values depend on the assay and the laboratory’s units of measurement. Overall, high values at T0 or a secondary rise by T2 should be regarded as warning flags for an unfavorable trajectory. Overall, our results indicate that renin is not only elevated in critical illness, but its temporal evolution carries additional prognostic information. We have indeed highlighted that patients with sustained or recurrent increases in renin during the ICU stay had worse clinical outcomes, including prolonged mechanical ventilation and higher mortality. These findings are consistent with recent reports linking renin dynamics to outcome in sepsis and ARDS (13, 20). These observed fluctuations in plasma renin concentrations can be interpreted in light of the pathophysiological alterations occurring in critically ill patients with COVID-19. Renin release is primarily stimulated by renal hypoperfusion, sympathetic nervous system activation, and reduced sodium delivery to the macula densa. In the context of critical illness, elevated renin concentrations therefore reflect impaired systemic and renal perfusion as well as microcirculatory dysfunction (21, 22). In our cohort, renin was elevated in nearly all patients at ICU admission, indicating an early and global activation of the renin–angiotensin–aldosterone system (RAAS). The initial decline in renin observed even among the most severely ill patients may represent a partial restoration of macrocirculatory parameters following aggressive early resuscitation. However, the subsequent rebound in renin among non-survivors suggests that the underlying pathophysiological mechanisms, such as persistent shock, ongoing inflammation, or endothelial injury, were not adequately controlled by standard interventions. This aligns with the concept that renin is not simply a marker of hemodynamic status at a single time point but rather integrates the burden of systemic and renal stress over time (23). The interpretation of renin as a disease-driven biomarker should be considered in the context of RAAS-modulating therapies. In our cohort, all ACE inhibitors and angiotensin receptor blockers were discontinued upon ICU admission, in line with early practices during the pandemic. While subsequent evidence has shown that these medications are safe for patients with COVID-19, their withdrawal may have impacted renin levels and their changes over time. Therefore, the renin trajectories observed in this study should be understood within this specific therapeutic context. It remains to be determined whether similar prognostic patterns are maintained in patients who continue receiving RAAS inhibition or in non-COVID ARDS populations where these therapies are typically continued. The temporal pattern observed for renin suggests a biphasic signal. Elevated renin levels early after ICU admission may primarily reflect baseline disease severity and acute circulatory stress at the onset of critical illness. In contrast, a secondary increase following an initial decline may indicate persistent or recurrent physiological stress during prolonged ICU stay. Although this later pattern could be influenced by complications or escalating organ dysfunction, the retrospective nature of the study and the lack of systematically recorded complication data preclude definitive attribution to specific clinical events. Therefore, renin trajectories should be interpreted as integrative markers of disease severity over time rather than as direct indicators of discrete complications.

This text provides a biological framework for understanding the prognostic findings of this study. Plasma renin reflects a combination of signals related to renal hypoperfusion, sympathetic activation, and the maladaptive response of the renin-angiotensin-aldosterone system (RAAS) (24, 25). This integrated signal can link early circulatory stress to persistent organ dysfunction and poor long-term outcomes. Evaluating renin trajectories has clinical significance, as it can identify patients at higher risk for unfavorable prognoses. The subsequent analyses will assess the predictive ability of renin in relation to established clinical indices and inflammatory markers. On univariate, one-variable-at-a-time logistic screening, several factors were associated with 1-year mortality: older age, higher Charlson, higher SAPS II and SOFA, a lower P/F ratio at T0, higher renin at T0 and T2, higher lactate and PCT at T2, lower MAP at T1, and vasopressor use at T2. Focusing on renin, the odds ratios per 1-SD were 1.94 at T0, 1.69 at T1 (*p* = 0.014), and 2.52 at T2 (all *p* ≤ 0.014). Taken together, these results indicate that the renin signal is already present early at T0 and becomes stronger again by T2, consistent with the hypothesized rebound pattern in non-survivors. In the multivariable logistic model restricted to complete cases, three predictors remained independently associated with 1-year mortality: a lower P/F ratio at T0, higher renin at T0, and higher PCT at T2, while the Charlson index showed a borderline association (*p* = 0.054). Although renin at T2 was strongly associated with outcome in univariate analyses, it did not retain independence after adjustment, likely to reflect overlap with late severity factors. Overall model discrimination was high: sensitivity and specificity were 0.92 and 0.88, respectively. In this context, it is essential to emphasize the role of PCT, an indicator of inflammation widely used in clinical practice, useful for stratifying prognostic risk in intensive care settings and historically used for the early identification of patients at high risk of developing septic shock (26). This study highlights that early plasma renin provides important prognostic information beyond traditional severity indices. In models accounting for respiratory impairment and overall illness severity, renin levels measured at 72 h remained independently associated with 1-year mortality. In contrast, scores like SOFA lost independent significance when overlapping factors were considered. This suggests that renin reflects a unique biological pathway linked to the activation of the renin-angiotensin-aldosterone system and ongoing circulatory and renal stress, rather than just acute organ dysfunction.

Therefore, renin should be viewed as an additional biomarker that enhances risk assessment when combined with standard indices. Integrating diverse analytical dimensions can improve the identification of high-risk patients, and the independent link between early renin and long-term mortality underscores its value in comprehensive prognostic frameworks (27, 28). Its prognostic role has also been confirmed in the subcategory of patients with COVID-19 infection. It allows the identification of patients at higher risk of severe infections and complications, which can lead to death, thus enabling the selection of patients who may be candidates for more aggressive therapies (29). In patients with COVID-19 infection, a close correlation has also been demonstrated between high PCT levels and the risk of bacterial superinfection, mainly from Gram-negative pathogens such as Pseudomonas or Acinetobacter. Superinfections are a major cause of death in these patients and could therefore explain the close correlation between high PCT levels and patient outcomes (30, 31). In our study, we demonstrated that high levels of PCT at T2 correspond to high levels of renin, and considering the above, these could be indicative of a septic superinfection.

In addition to this, renin may provide complementary information to other biomarkers routinely used in ICU settings. While lactate is a marker of global tissue hypoxia and procalcitonin or interleukin-6 (IL-6) are markers of inflammation, renin appears to capture a distinct dimension of critical illness, namely the maladaptive activation of RAAS (32). In this respect, renin may represent an early indicator of “renal distress” preceding overt acute kidney injury, as previously proposed. Moreover, since all patients had ACEi/ARB therapy suspended upon ICU admission, the renin trajectory observed in our study is likely attributable to the severity of the disease process itself rather than to pharmacological interference. Chronic ACEi/ARB use was neither different at baseline between outcome groups nor associated with 1-year mortality in univariate or multivariable analyses; moreover, all chronic ACEi/ARB therapies were suspended on ICU admission, limiting pharmacologic confounding on renin trajectories. Taken together, these findings support the interpretation of renin as a dynamic biomarker of circulatory failure and systemic stress, which might help identify patients at risk of deterioration despite apparently stable macrocirculatory parameters. In COVID-19, renin occupies a distinct pathophysiological niche compared with commonly used biomarkers and therefore adds complementary prognostic information. Inflammatory markers such as CRP and IL-6 primarily reflect cytokine activation and correlate with systemic inflammation, while PCT is more useful for antimicrobial stewardship than for long-term risk stratification (33–35). Pancreatic Stone Protein (PSP) sometimes referred to as pancreatic inflammatory protein, captures a related but not identical axis of host response: it is a stress-responsive secretory protein (of pancreatic/intestinal origin) that tracks infection-induced innate immune activation and, in several ICU cohorts, rises early and dynamically with emerging sepsis, in some cases outperforming PCT for early detection (36–39). Coagulation markers (e.g., D-dimer) reflect the thrombo-inflammatory phenotype of severe COVID-19, and cardiac injury markers (troponin, NT-proBNP) index myocardial stress. By contrast, renin integrates the hemodynamic and renal dimensions of critical illness, mirroring RAAS activation in response to impaired perfusion and sympathetic drive (40). In our cohort, early renin (T0) remained independently associated with one-year mortality even after accounting for respiratory impairment (P/F) and inflammation (PCT), indicating prognostic signal not shared by these axes. Practically, assay dependence (plasma renin activity [PRA] vs. PRC) argues for laboratory-specific thresholds, standardized per-SD effects, and log-transformed analyses. Overall, positioning renin alongside inflammatory (CRP/IL-6/PCT/PSP), coagulation (D-dimer), and organ-injury markers provides a more orthogonal view of COVID-19 pathobiology, supporting integration into multiparametric risk models and serial monitoring of treatment response.

Our study confirms the promising role of renin as a biomarker in critical care medicine, and for the first time provides data on association between dynamic renin changes and long-term outcome (13). In particular, our data suggest that an increase in renin >20% is associated with worse prognosis. These findings may be particularly interesting considering recent evidence highlighting limitations of current resuscitation targets, including mean arterial pressure (41) (MAP), and lactate (42, 43). Notably, studies suggest that renin may also be used to individualize vasopressor therapy in hypotensive patients, and may be used to guide multimodal vasopressor therapy (44–47). Measuring renin should be regarded as a complementary tool rather than the sole factor in treatment decisions. Due to variability in assay results and the absence of universally accepted cut-off values, its clinical usefulness is better gauged through relative changes over time. This study demonstrates that early elevations in renin, followed by a secondary increase after an initial decline, can help identify patients at a higher long-term risk. This suggests that interpreting renin levels based on their trajectory may be more informative. These comparisons aim to contextualize renin within the broader landscape of biomarkers in critical illness, while the primary focus of this study is on renin trajectories and their association with outcomes. Therefore, renin could be integrated into existing ICU risk assessment frameworks as an additional biomarker, alongside respiratory indices, global severity scores, and inflammatory markers. This integration may help identify patients experiencing ongoing circulatory or renal stress, even when macrocirculatory parameters appear stable, leading to closer monitoring or more intensive support. Future studies are needed to define actionable thresholds and evaluate whether management guided by renin levels improves outcomes.

### Strengths and limitations

This study is retrospective and single-center, focused on mechanically ventilated ICU patients with COVID-19, so results should be interpreted in the context of local practice and may not generalize to all settings. Nevertheless, our results are consistent with data obtained from other populations (12). Although 249 patients were admitted during the period, the main analyses used 104 patients with serial biomarkers and the multivariable model was based on 86 complete cases; nonetheless, effect directions were coherent across analyses. Renin was measured as plasma renin concentration at three pragmatic timepoints (72, 120, 168 h) determined by assay availability, which supports feasibility but does not capture fully continuous dynamics; to enhance comparability, we reported laboratory-specific tertiles, standardized per-SD effects, and log-transformed analyses. An important limitation of our study relates to the management of renin-angiotensin-aldosterone system (RAAS) inhibitors. In our cohort, all chronic ACE inhibitor and angiotensin receptor blocker therapies were systematically discontinued upon admission to the ICU, in line with institutional practices during the early phase of the COVID-19 pandemic, when there were concerns about their safety. Subsequent evidence has shown that these agents are safe and do not worsen outcomes in patients with COVID-19. Therefore, the systematic discontinuation of these medications in our study may have affected renin levels and their temporal trajectories. As a result, the observed association between elevated renin levels and mortality may not be directly applicable to patients whose ACE inhibitors or angiotensin receptor blockers are continued or reintroduced during critical illness. This consideration also extends to ARDS of non-COVID-19 etiology, where the management of RAAS-modulating therapies may differ. Future studies are needed to determine whether the prognostic significance of renin is maintained when pharmacological RAAS modulation is ongoing. A further limitation is that Ang II and aldosterone levels were not measured, because these assays were not available in our laboratory at the time of the study. Therefore, we cannot determine whether elevated renin reflected an appropriate compensatory response to preserve blood pressure and tissue perfusion, or a maladaptive state of sustained RAAS activation potentially contributing to inflammation, endothelial dysfunction, thrombosis, and ischemic injury. In addition, exogenous Ang II was not available in our clinical setting during the study period, and renin-guided therapeutic strategies could not be evaluated. Future studies should assess renin together with downstream RAAS mediators, including Ang II and aldosterone, to clarify whether high-renin phenotypes identify patients who may benefit from continuation or interruption of RAAS blockade, or from exogenous Ang II in selected shock states. Data on clinical complications, including secondary infections during ICU stays, were not systematically available, limiting their inclusion in analyses. Procalcitonin was used as a surrogate marker for infectious burden, but future studies with detailed clinical and microbiological data are needed to clarify the relationship between renin trajectories, complications, and clinical deterioration. Data on ICU course variables, such as mechanical ventilation duration and hospital length of stay, were inconsistently available, representing a study limitation. In the context of COVID-19, prolonged hospitalizations and post-acute sequelae, like long COVID, can impact long-term outcomes, making 1-year mortality a meaningful endpoint encompassing both acute and post-acute phases of the disease. Kaplan–Meier curves at T1–T2 rely on post-baseline values and are provided as descriptive visualization, while the strong separation already at T0 supports an early prognostic signal. Although model discrimination was high (AUC 0.941), this performance should be interpreted with caution. The multivariable model was developed in a single-center cohort with a relatively limited sample size, and no optimism correction or internal validation (e.g., bootstrapping or cross-validation) was performed. As such, the reported AUC may overestimate true predictive performance, and external validation in independent, multicenter cohorts is required before clinical implementation. Together, these considerations temper but do not diminish the central finding that early (T0) renin and its trajectory convey clinically meaningful prognostic information in severe COVID-19.

## Conclusion

Our study suggests that in patients with severe COVID-19 disease, early renin levels are associated with 1-year outcome. Furthermore, a late rebound in levels after an initial decline is also associated with worse 1-year survival. These associations were observed independently of chronic renin–angiotensin system inhibitor therapy, which was discontinued on ICU admission, supporting a disease-driven rather than pharmacological interpretation of renin dynamics. Large multicenter studies are warranted to confirm these findings in other settings, and to assess whether interventions based on renin assessment could improve outcome in critically ill patients.

## Data Availability

The raw data supporting the conclusions of this article will be made available by the authors, without undue reservation.
