# Supplementary figures and images for: Renin and 1-year mortality in critically ill patients with ARDS: trajectories, discrimination, and survival analysis
Source: Front Med (Lausanne). 2026 Jun 23;13:1806797. doi: 10.3389/fmed.2026.1806797 (PMC13337720; doi:10.3389/fmed.2026.1806797)

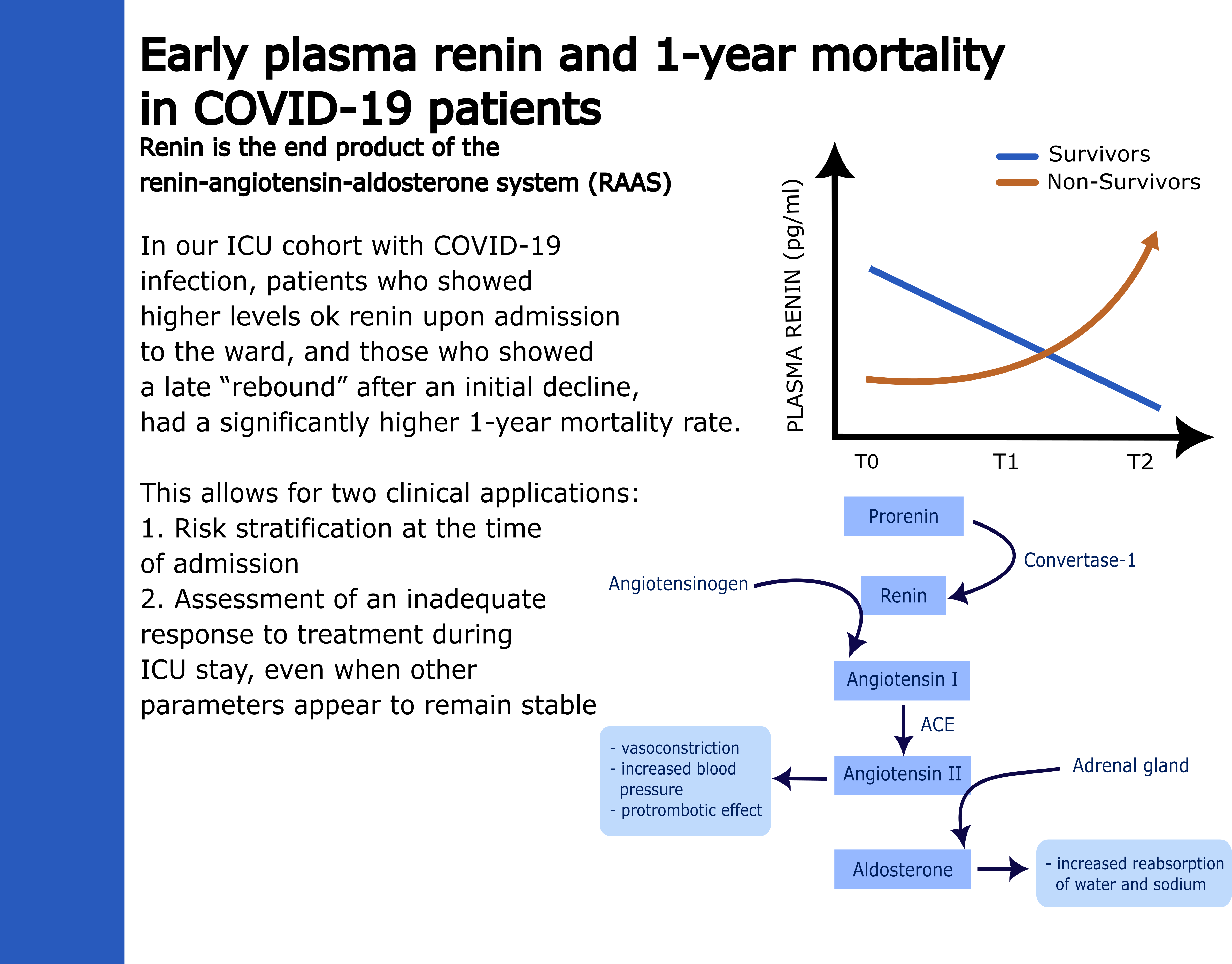

Supplement: Supplementary file 1 [file Image_1.PNG]
