# Supplementary material for: Renin and 1-year mortality in critically ill patients with ARDS: trajectories, discrimination, and survival analysis
Source: Front Med (Lausanne). 2026 Jun 23;13:1806797. doi: 10.3389/fmed.2026.1806797 (PMC13337720; doi:10.3389/fmed.2026.1806797)
Supplement: Supplementary file 2 [file Table_1.DOCX]

**Supplementary** material

| Variabile | N | Pearson r | p-value |
| --- | --- | --- | --- |
| Renin T0 | 104 | 0.311 | 0.0013 |
| Renin T1 | 104 | 0.248 | 0.0111 |
| Renin T2 | 104 | 0.402 | <0.0001 |
| Lactate T0 | 104 | 0.132 | 0.1829 |
| Lactate T1 | 104 | 0.197 | 0.0455 |
| Lactate T2 | 104 | 0.308 | 0.0015 |
| PCT T0 | 97 | 0.128 | 0.2102 |
| PCT T1 | 102 | 0.200 | 0.0442 |
| PCT T2 | 103 | 0.340 | 0.0004 |

Pearson correlations were computed between 1-year mortality (binary outcome coded 0/1) and log(1+x) transformed biomarkers; p-values are two-sided.

SOFA score was analyzed as a continuous outcome. Separate univariable linear regression models were fitted for each biomarker and timepoint. Biomarkers were log(1+x) transformed due to right-skewed distributions. β coefficients represent the expected change in SOFA score per unit increase in the log-transformed biomarker.

| Predictor | N | β coefficient | 95% CI | p-value |
| --- | --- | --- | --- | --- |
| Renin T0 | 104 | 0.742 | 0.328 – 1.156 | 0.0006 |
| Lactate T0 | 104 | 3.012 | 1.126 – 4.899 | 0.0021 |
| PCT T0 | 97 | 0.621 | −0.312 – 1.554 | 0.191 |

| Predictor | N | β coefficient | 95% CI | p-value |
| --- | --- | --- | --- | --- |
| Renin T1 | 104 | 0.564 | 0.177 – 0.952 | 0.0045 |
| Lactate T1 | 104 | 2.144 | 0.214 – 4.073 | 0.0297 |
| PCT T1 | 102 | 1.479 | 0.455 – 2.503 | 0.0051 |

| Predictor | N | β coefficient | 95% CI | p-value |
| --- | --- | --- | --- | --- |
| Renin T2 | 104 | 0.816 | 0.378 – 1.254 | 0.00036 |
| Lactate T2 | 104 | 5.309 | 2.761 – 7.856 | <0.0001 |
| PCT T2 | 103 | 2.234 | 1.027 – 3.441 | 0.00039 |

Thirty-day mortality was defined as death within 30 days from ICU admission (Decesso 1 anno = 1 and Time Surv ≤ 30). Univariable logistic regression models were fitted for each biomarker and timepoint. Biomarkers were analyzed as log(1+x) and standardized; odds ratios are reported per 1 SD increase.

| Predictor (log(1+x), per 1 SD) | N | OR | 95% CI | p-value |
| --- | --- | --- | --- | --- |
| Renin T0 | 104 | **1.84** | 1.23–2.75 | **0.0029** |
| Renin T1 | 104 | **1.58** | 1.07–2.35 | **0.0217** |
| Renin T2 | 104 | **2.00** | 1.37–2.93 | **0.0003** |
| Lactate T0 | 104 | 1.43 | 0.94–2.16 | 0.0963 |
| Lactate T1 | 104 | **1.55** | 1.00–2.40 | **0.0475** |
| Lactate T2 | 104 | **1.83** | 1.18–2.85 | **0.0074** |
| PCT T0 | 97 | 1.25 | 0.83–1.89 | 0.2783 |
| PCT T1 | 102 | 1.41 | 0.94–2.11 | 0.0929 |
| PCT T2 | 103 | **1.63** | 1.07–2.48 | **0.0222** |

**Timepoint contrasts from log-quadratic models**

For each biomarker (Renin, Lactate, CRP, PCT) and timepoint (72 h, 120 h, 168 h), the table reports the adjusted log-difference (Non-survivors − Survivors), robust SE, Wald p-value, and the geometric mean ratio with patient-level bootstrap 95% CI.

| Biomarker | Time (h) | Log‑diff (NS − S) | SE (robust) | p‑value | Ratio (NS/S) | 95% CI (ratio) |
| --- | --- | --- | --- | --- | --- | --- |
| Renin | 72 | 0.892 | 0.272 | 0.001 | 2.440 | 1.457–4.108 |
| Renin | 120 | 0.670 | 0.255 | 0.009 | 1.955 | 1.203–3.106 |
| Renin | 168 | 1.121 | 0.253 | 0.000 | 3.068 | 1.783–5.081 |
| Lactate | 72 | 0.094 | 0.071 | 0.186 | 1.099 | 0.943–1.264 |
| Lactate | 120 | 0.079 | 0.039 | 0.041 | 1.082 | 1.009–1.165 |
| Lactate | 168 | 0.146 | 0.045 | 0.001 | 1.157 | 1.042–1.262 |
| CRP | 72 | 0.126 | 0.155 | 0.417 | 1.134 | 0.876–1.497 |
| CRP | 120 | 0.302 | 0.170 | 0.075 | 1.353 | 0.972–1.832 |
| CRP | 168 | 0.400 | 0.189 | 0.035 | 1.491 | 1.066–2.129 |
| PCT | 72 | 0.173 | 0.138 | 0.210 | 1.189 | 0.838–1.551 |
| PCT | 120 | 0.244 | 0.123 | 0.047 | 1.277 | 1.012–1.603 |
| PCT | 168 | 0.347 | 0.099 | 0.000 | 1.415 | 1.162–1.684 |
